# Predictiveness curves in virtual screening

**DOI:** 10.1186/s13321-015-0100-8

**Published:** 2015-11-04

**Authors:** Charly Empereur-mot, Hélène Guillemain, Aurélien Latouche, Jean-François Zagury, Vivian Viallon, Matthieu Montes

**Affiliations:** Laboratoire Génomique Bioinformatique et Applications, EA 4627, Conservatoire National des Arts et Métiers, 292 rue Saint Martin, 75003 Paris, France; Equipe MSDMA, Laboratoire CEDRIC, EA 4629, Conservatoire National des Arts et Métiers, 292 rue Saint Martin, 75003 Paris, France; Université de Lyon, 69622 Lyon, France; UMRESTTE, Université Lyon 1, 69373 Lyon, France; UMRESTTE, IFSTTAR, 69675 Bron, France

## Abstract

**Background:**

In the present work, we aim to transfer to the field of virtual screening the predictiveness curve, a metric that has been advocated in clinical epidemiology. The literature describes the use of predictiveness curves to evaluate the performances of biological markers to formulate diagnoses, prognoses and assess disease risks, assess the fit of risk models, and estimate the clinical utility of a model when applied to a population. Similarly, we use logistic regression models to calculate activity probabilities related to the scores that the compounds obtained in virtual screening experiments. The predictiveness curve can provide an intuitive and graphical tool to compare the predictive power of virtual screening methods.

**Results:**

Similarly to ROC curves, predictiveness curves are functions of the distribution of the scores and provide a common scale for the evaluation of virtual screening methods. Contrarily to ROC curves, the dispersion of the scores is well described by predictiveness curves. This property allows the quantification of the predictive performance of virtual screening methods on a fraction of a given molecular dataset and makes the predictiveness curve an efficient tool to address the early recognition problem. To this last end, we introduce the use of the total gain and partial total gain to quantify recognition and early recognition of active compounds attributed to the variations of the scores obtained with virtual screening methods. Additionally to its usefulness in the evaluation of virtual screening methods, predictiveness curves can be used to define optimal score thresholds for the selection of compounds to be tested experimentally in a drug discovery program. We illustrate the use of predictiveness curves as a complement to ROC on the results of a virtual screening of the Directory of Useful Decoys datasets using three different methods (Surflex-dock, ICM, Autodock Vina).

**Conclusion:**

The predictiveness curves cover different aspects of the predictive power of the scores, allowing a detailed evaluation of the performance of virtual screening methods. We believe predictiveness curves efficiently complete the set of tools available for the analysis of virtual screening results.

**Electronic supplementary material:**

The online version of this article (doi:10.1186/s13321-015-0100-8) contains supplementary material, which is available to authorized users.

## Background

Structure-based and ligand-based virtual screening of compound collections has become extensively used in drug discovery programs to reduce the number of compounds going into high throughput screening procedures [1]. The aim of virtual screening methods is to enrich a subset of molecules in potentially active compounds while discarding the compounds supposed to be inactive according to a scoring function [2]. One of the issues with their use in prospective screening is to choose an optimal score selection threshold for experimental testing. It is usually estimated empirically through the analysis of retrospective virtual screening outputs on benchmarking datasets, which include known active compounds and putative inactive compounds (also known as decoys).

In this context, different metrics have emerged to evaluate the performance of virtual screening methods: enrichment factors (EFs), receiver operating characteristics (ROC) curves [2], the area under the ROC curve (ROC AUC) [2], the partial area under the ROC curve (pAUC) [3], the Boltzmann-enhanced discrimination of ROC (BEDROC) [4], the robust initial enhancement (RIE) [5]; ROC and EF being the most widely used. The ROC curves and their AUC provide a common scale to compare the performances of virtual screening methods. However, the ROC curves and their AUC suffer from two limitations. First, virtual screening methods are used to prioritize a subset of the screened compound collection for experimental testing, whereas ROC curves and ROC AUC summarize the ability of a method to rank a database over its entirety [4, 6]. Second, these two metrics are exclusively based on the ranks obtained by the compounds according to the score they obtained with the virtual screening method and do not take into account the difference in score between successively ranked compounds. Additionally, ROC curves are not suited to estimate the size of the molecular fraction selected at a given threshold. The true positive fraction (TPF) and false positive fraction (FPF) of the ROC plot can reflect a very different number of compounds on an identical scale, which can be misleading for analyzing the early recognition of active compounds.

EFs are more reliable towards the early recognition problem, since they are focused on the true positive fraction [2]. However, with EFs, the “ranking goodness” before the fractional threshold is not taken into account and their maximum value is strongly dependent on the ratio of active compounds over decoys in the benchmarking dataset (i.e. prevalence of activity) [2, 4, 7]. Another problem reported in previous studies is that metrics that seem to be statistically different such as ROC AUC, BEDROC, the area under the accumulation curve (AUAC) and the average rank of actives are in fact intimately related [4, 7, 8].

Different metrics have been proposed to overcome the limitations of the widely used EF and ROC curves, such as pAUC [3], BEDROC [4] and RIE [5], which better address early recognition. However, some limitations still persist: (1) the rank-based problems of ROC AUC are inherited by pAUC; (2) the maximum RIE value is dependent on the ratio of active compounds over decoys (similarly to EFs) [4]; and 3. BEDROC is dependent on a single parameter that embodies its overall sensitivity and that has to be selected according to the importance given to the early ranks. Unbiased comparisons between different evaluations are then rendered difficult by such a sensitive parameter [4, 6].

In the present work, we aimed to transfer to the field of virtual screening the Predictiveness Curve (PC) [9], a metric that has already been advocated in clinical epidemiology [10–14], where the values of biomarkers are used to formulate diagnoses, prognoses and assess disease risks. The use of PCs is described in the literature to evaluate the performance of given biological markers, to assess the fit of risk models and to estimate the clinical utility of a model when applied to a population. The dispersion of the scores attributed to the compounds by a given method is emphasized with the predictiveness curve, providing complementary information to classical metrics such as ROC and EF. Predictiveness curves can be used to (1) quantify and compare the predictive power of scoring functions above a given score quantile; and (2) define a score threshold for prospective virtual screening, in order to select an optimal number of compounds to be tested experimentally in a drug discovery program. In this study, we show how PCs can be used to graphically assess the predictive capacities of virtual screening methods, especially useful when considering the early recognition problem. Next, we applied the PC to the analysis of retrospective virtual screening results on the DUD database [15] using three different methods: Surflex-dock [16], ICM [17], and Autodock Vina [18]. We introduced the use of the total gain (TG) [19] to quantify the contribution of virtual screening scores to the explanation of compound activity. Standardized TG (noted as TG) ranges from 0 (no explanatory power) to 1 (“perfect” explanatory power) and can be visualized directly from the predictiveness curve [19]. Similarly, the partial total gain (pTG) [20] allows the explanatory power of virtual screening scores in the early part of the benchmarking dataset to be quantified as a partial summary measure of the PC. By monitoring the performances of three virtual screening methods using the predictiveness curve, TG and pTG on the DUD dataset, we have proposed a new approach to define optimal score thresholds adjusted to each target. Finally, we have discussed the interests of using predictiveness curves, total gain and partial total gain in addition to the ROC curves to better assess the performances of virtual screening methods and optimize the selection of compounds to be tested experimentally in prospective studies.

## Methods

### The directory of useful decoys (DUD) dataset

The DUD is a public benchmarking dataset designed for the evaluation of docking methods containing known active compounds for 40 targets, including 36 decoys for each active compound [15]. We selected for each target its corresponding DUD-own dataset that comprises only its associated active compounds and decoys. In our study, we used DUD release 2 dataset available at http://dud.docking.org.

### Selection and preparation of the protein structures

We selected for this study the 39 targets issued from the DUD for which at least one experimental structure was available. Target PDGFR-β was thus excluded since it was obtained through homology modeling. Hydrogen atoms were added using Chimera [21].

### Computational methods

#### Surflex-dock

Surflex-dock is based on a modified Hammerhead fragmentation-reconstruction algorithm to dock compound flexibly into the binding site [16]. The query molecule is decomposed into rigid fragments that are superimposed to the Surflex protomol (i.e. molecular fragments covering the entire binding site). The docking poses were evaluated by an empirical scoring function. For each structure, the binding site was defined at 4Å around the co-crystallized ligand for the protomol generation step. In this study, Surflex-dock version 2.5 was used for all calculations.

#### ICM

ICM is based on Monte Carlo simulations in internal coordinates to optimize the position of molecules using a stochastic global optimization procedure combined with pseudo-Brownian positional/torsional steps and fast local gradient minimization [17]. The docking poses were evaluated using the ICM-VLS empirical scoring function [22]. The binding sites defined for docking were adjusted to be similar to the Surflex protomol. ICM version 3.6 was used for all calculations.

#### AutoDock Vina

Autodock Vina generates docking poses using an iterated local search global optimizer [23] which consists in a succession of steps of stochastic mutations and local optimizations [18]. At each step, the Broyden-Fletcher-Goldfarb-Shanno algorithm (BFGS) is used for local optimization [24]. Autodock Vina evaluated docking poses using its own empirical scoring function. The binding sites have been defined identically to the ones used for Surflex-dock and ICM calculations to obtain similar spatial search areas in all of the docking experiments. We used Autodock Vina version 1.1.2 for all calculations.

### ROC curves analysis

The ROC curve applied to the retrospective analysis of a virtual screening experiment is a plot of the true positive fractions (TPF, y-axis) versus false positive fractions (FPF, x-axis) for all compounds in a ranked dataset [2, 6]. Each point of the ROC curve then represents a unique TPF/FPF pair corresponding to a particular fraction of the molecular dataset. A scoring function that would be able to perform perfect discrimination (i.e. no overlap between the two distributions of active and inactive compounds according to their calculated scores of binding affinity) has a ROC curve that passes through the upper left corner of the plot, where the TPF is 1 (perfect sensitivity) and the FPF is 0 (perfect specificity). The theoretical ROC curve resulting from an experiment in which the scoring function would have no discrimination is a 45° diagonal line from the lower left corner to the upper right corner. Qualitatively, the closer the curve is to the upper left corner, the higher the overall accuracy of the test. The area under the ROC curve (ROC AUC) summarizes the overall performance of a virtual screening experiment [2], whereas the partial area under the ROC curve (pAUC) allows to focus on a specific region of the curve and is usually calculated at a given early FPF value [3].

### Predictiveness curves calculation

The approach we used in this study relies on the use of logistic regression to model how the scores issued by virtual screening methods explain the activity of the compounds in a virtual screening experiment. We used generalized linear models with a binomial distribution function and the canonical log link to calculate each compound probability of activity from the scores obtained by the compounds in a virtual screening experiment. Parameters were fit using the iteratively reweighted least squares algorithm. The predictiveness curve was then built as a cumulative distribution function (CDF) of activity probabilities. Let *A* denote a binary outcome termed compound activity where *A* = 1 for active and *A* = 0 for inactive. The probability of a compound to be active given its VS score *Y* = *y* is *P*_act_(y) = *P*[*A* = 1 | *Y* = *y*]. We proposed the use of the predictiveness plots, *R*(*v*) versus *v*, to describe the predictive capacity of a VS method, where *R*(*v*) is the activity probability associated with the *v*th quantile of the VS scores: *R*(*v*) = *P*[*A* = 1 | *Y* = *F*^−1^(*v*)], and *F* is the CDF of VS scores. Hence, predictiveness plots provide a common scale for making comparisons between VS methods that may not be comparable on their original scales [12]. Suppose *p*_*L*_ and *p*_*H*_ are two thresholds that define “low probability of activity” and “high probability of activity”. Then the proportions of the compounds with low, high, and equivocal probabilities of activity are *R*^−1^(*p*_*L*_), 1 − *R*^−1^(*p*_*H*_) and *R*^−1^(*p*_*H*_) − *R*^−1^(*p*_*L*_), respectively, using the inverse function of *R*(*v*). Virtual screening scores that are uninformative about compound activity assign equal activity probabilities to all compounds, *P*_act_(Y) = P[A = 1 | *Y*] = P[A = 1] = *p*, where *p* is the prevalence of activity in the molecular dataset. On the other hand, perfect VS scores assign *P*_act_(Y) = 1 for the proportion *p* of compounds with A = 1 and *P*_act_(Y) = 0 for the proportion 1 − *p* with A = 0. Correspondingly, its PC is the step function *R*(*v*) = *I*[(1 − *p*) < *v*], where *I* is the indicator function. Most scoring functions are imperfect, yielding activity probabilities between these extremes. Good predictions issued from virtual screening methods yield steeper predictiveness curves corresponding to wider variations of activity probabilities.

### Predictiveness plots analysis

**Fig. 1:**
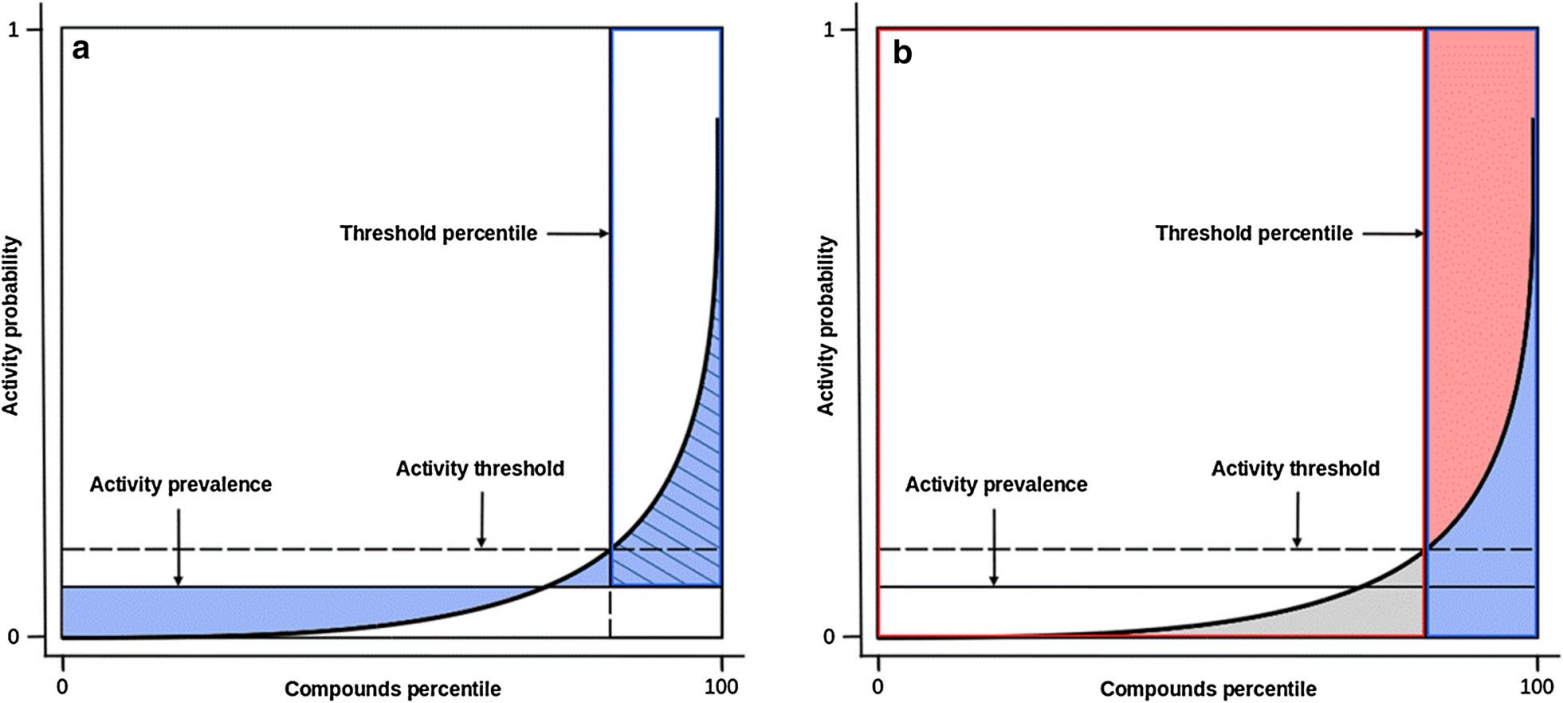
Schematic diagram presenting how performance metrics relate to the predictiveness curve. Displaying the PC allows for an intuitive selection of thresholds. Performance metrics related to a chosen threshold are easily interpreted from the curve. **a** Partial total gain (pTG): hatched area/*blue* frame; total gain (TG): *blue* area. **b** True positive fraction (TPF): *blue* area/area under activity prevalence; false positive fraction (FPF): *red* area/(1-area under activity prevalence); positive predictive value (PPV): *blue* area/*blue* frame; negative predictive value (NPV): *white area*/*red* frame

The ability of the models to highlight score gaps between compounds and relate those differences to activity probabilities allowed us to quantify the predictive power of virtual screening methods in terms of both scoring and ranking. Displaying the PC then allows for an intuitive analysis of the performances of virtual screening methods. The visualization of the total gain, partial total gain and the size of the molecular subset enables a straightforward interpretation of the results (Fig. 1a). For a completely uninformative model the PC would correspond to a horizontal line at the level of activity prevalence (Fig. 1). Inversely, steep predictiveness curves enable the observation of an inflexion point from which the curve rises. Hence, additionally to its benchmarking interests, PC provides a guidance to choose an optimal score threshold from VS results, allowing one to assess decision criteria from multiple points of view. Visualizing the curve allows to determine if activity probability variations are important enough to induce the selection of a threshold for prospective virtual screenings. Usual metrics can also be interpreted from the predictiveness curve: the true positive fraction (TPF), false positive fraction (FPF), positive predictive value (PPV) and negative predictive value (NPV) (Fig. 1b).

### Performance metrics

Statistical analysis was conduced using the R software [25]. The package ROCR [26] was used to plot ROC curves and perform ROC and partial ROC AUC calculations.

Enrichment factors were computed as follows:

$$EF_{x\% } = \frac{{Hits_{x\% } /N_{x\% } }}{{Hits_{t\% } /N_{x\% } }}$$

where *Hits*_*x%*_ is the number of active compounds in the top *x%* of the ranked dataset, *Hits*_*t*_ is the total number of active compounds in the dataset, *N*_*x%*_ is the number of compounds in the *x%* of the dataset and *N*_*t*_ is the total number of compounds in the dataset.

The contribution of virtual screening scores to the explanation of compounds activity can be quantified over a dataset using the standardized total gain (TG) [19], introduced by Bura et al. as a summary measure of the predictiveness curve:

$$\overline{TG} (v) = \frac{{\int_{0}^{1} {\left| {R(v) - p} \right|} \,dv}}{2p\,(1 - p)}$$

where *p* is the prevalence of activity in the molecular dataset and *R*(*v*) is the value of the activity probability at the *v*th quantile. The total gain is normalized by its maximum value, so that TG values are in the range [0,1] (null to perfect explanatory power). TG summarizes the proportion of variance in a binomial outcome explained by the model. In our application, TG quantifies the success of a VS method to rank and score compounds depending on activity, over the complete molecular dataset.

The predictive performance of VS scores can be quantified above the *v*th quantile of the molecular dataset using the partial total gain (pTG) [20], recently introduced by Sachs et al. as a partial summary measure of the PC, defined as:

$$pTG(v) = \frac{{\int_{v}^{1} {\left| {R(v) - p} \right|} \,dv}}{(1 - v)(1 - p)}$$

where *p* is the prevalence of activity in the molecular dataset and *R*(*v*) is the value of the activity probability at the *v*th quantile of the dataset. The denominator term is a standardization factor leading to pTG values in the range of 0 to 1 and makes pTG prevalence independent. pTG summarizes the proportion of variance in a binomial outcome explained by the model above the *v*th quantile. In our application, pTG quantifies the contribution of virtual screening scores to the explanation of compounds activity above the *v*th quantile of the molecular dataset.

## Results

### Assessment of the predictive power of a scoring function

**Fig. 2:**
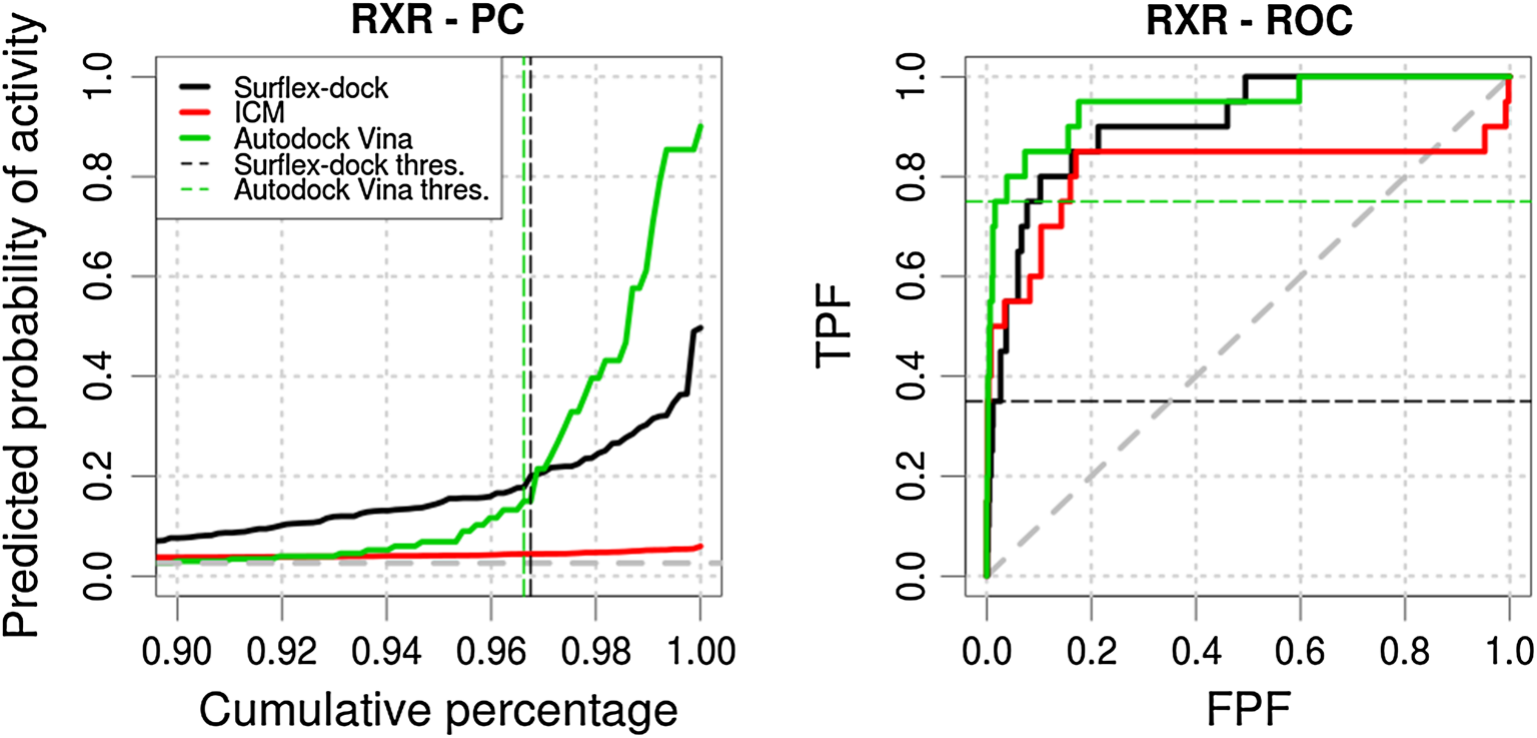
Predictiveness and ROC curves for the virtual screenings of ACE, ACHE, ADA, ALR2, AMPC, AR, CDK2, COMT, COX1 and COX2 selected from the DUD datasets using Surflex-dock, ICM and Autodock Vina (*black*, *red* and *green curves*, respectively). *Dashed gray lines* indicate the prevalence of activity and random picking of compounds. *Vertical dashed lines* represent the thresholds we manually selected from the analysis of the curves. Metrics associated to the selected thresholds are available in Tables 2, 3, 4. Partial metrics at 2 % and 5 % of the ranked dataset are available in Additional file 1: Table S1; Additional file 2: Table S2 and Additional file 3: Table S3

We first illustrated the use of the predictiveness curve as a complement to the ROC curve with the results obtained from Surflex-dock, ICM, and Autodock Vina on target retinoic X receptor (RXR) of the DUD dataset (Fig. 2). For these methods, the ROC AUCs indicated that the discrimination of active compounds over inactive compounds within the complete dataset was successful (Surflex-dock: 0.907, ICM: 0.812, Autodock Vina: 0.944). The ROC curve profiles suggested that acceptable early recognition has been achieved by the three methods (Surflex-dock pAUC2 %: 0.167, ICM pAUC2 %: 0.342, Autodock Vina pAUC2 %: 0.330), which was confirmed in terms of enrichment (Surflex-dock EF2 %: 16.84, ICM EF2 %: 24.06, Autodock Vina EF2 %: 26.47). Under these conditions, following the first described use of the ROC curves for the analysis of virtual screening results [2], score selection thresholds could be extracted from the curve points prior to FPF = 0.2 by maximizing the sensitivity or the specificity of the method.

In the present case, the analysis of the predictiveness curves brought complementary insights. Total gain values indicated that the detection of the activity of the compounds is related to more important score variations with Autodock Vina, compared to ICM and Surflex-dock (Surflex-dock TG = 0.675, ICM TG = 0.124, Autodock Vina TG = 0.740). The contributions of each scoring function to the early detection of active compounds can be quantified using the partial total gain (Surflex-dock pTG2 %: 0.308, ICM pTG2 %: 0.026, Autodock Vina pTG2 %: 0.653), which enables a straightforward comparison of the performances of the methods in a limited range of the dataset. In the case of ICM, even if the ROC curve profile supported that global and early enrichments are achieved, the associated PC corresponded to a *quasi* null-model, associated to a low TG value. Even if ICM was able to rank the active compounds satisfactorily, the analysis of the PC informed us that the score variations between the active compounds and the decoys were not representative of the activity of the compounds. Then, deriving score thresholds from the analysis of retrospective virtual screening experiments with ICM would not be relevant for the prospective detection of active compounds on RXR.

The PCs could graphically emphasize the performance of each method on early enrichment, highlighting that the most predictive method towards the activity of the compounds on RXR was Autodock Vina, over Surflex-dock and ICM.

### Selection of optimal score thresholds

A visual analysis of the PCs for RXR clearly displayed that Autodock Vina outperformed Surflex-dock and ICM in terms of early enrichment and that its scoring function would be more predictive of activity within its high scores. In particular, for Autodock Vina on this target, an inflexion point was observable where the PC rose steeply (3.38 % of the ranked dataset), which allowed the retrieval of a score selection threshold from which the scores are highly associated with the activity of the compounds in the corresponding subset (Autodock Vina pTG3.38 %: 0.488, Autodock Vina EF3.38 %: 21.39) (Fig. 2, vertical dashed green line). The pTG of 0.488 in the selected subset signified that each compound in this subset has an average probability gain of 0.488 of being active over the random picking of compounds. For Surflex-dock the PC showed a different profile, gradually increasing to reach activity probabilities over 0.5. In this particular case, the threshold selection is graphically estimated depending on the size of the selected subset. We have estimated the optimal selection threshold for Surflex-dock at 3.25 % of the ranked dataset (Surflex-dock pTG3.25 %: 0.265, Surflex-dock EF3.25 %: 10.37) (Fig. 2, vertical dashed black line), which was close to the optimal threshold retrieved with Autodock Vina. We then projected these two thresholds on the ROC curves (Fig. 2, horizontal colored dashed lines). Interestingly, the visualization of these two thresholds on the PC and ROC curves emphasized the bias induced by the ROC towards the estimation of the size of the selected subset. For the two close selected thresholds the corresponding points on the ROC curves largely differ emphasizing that the ROC curves are not adapted to visualize the size of the selected datasets (Surflex-dock TPF3.25 %: 0.350, Surflex-dock FPF3.25 %: 0.025, Autodock Vina TPF3.38 %: 0.750, Autodock Vina FPF3.38 %: 0.016).

### Emphasize on the different early recognition profiles

We performed virtual screening experiments on 39 targets from the DUD dataset using Surflex-dock, ICM and Autodock Vina. For 9 out of the 39 targets (ACHE, AMPC, FGFR1, GR, HIVRT, HSP90, PR, TK and VEGFR2), none of the three virtual screening methods yielded differences in score that were predictive of the activity of the compounds, resulting in PCs *quasi* null-model profile and very low TG values.

**Fig. 3:**
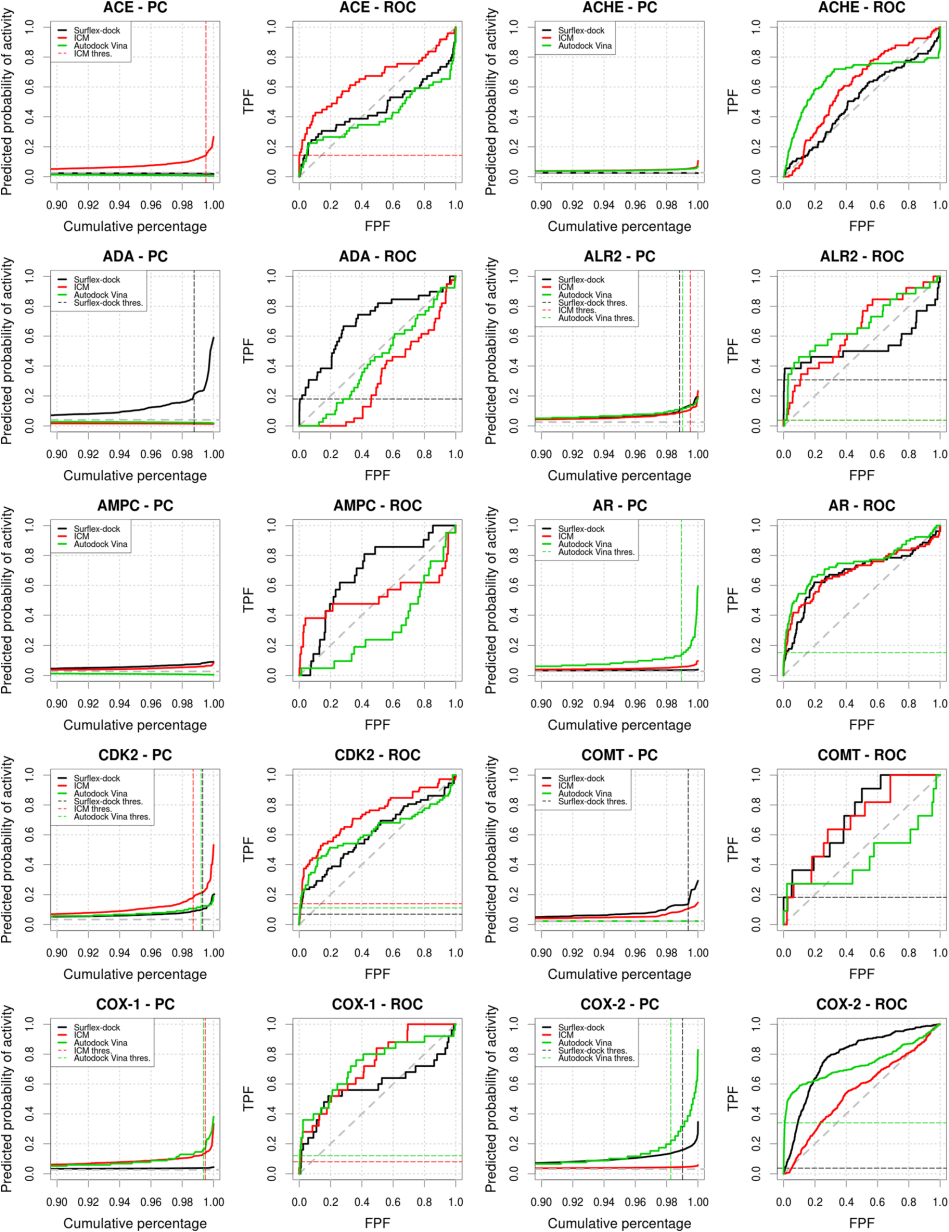
Predictiveness and ROC curves for the virtual screenings of DHFR, EGFR, ER, FGFR1, FXA, GART, GPB, GR and HIVPR selected from the DUD datasets using Surflex-dock, ICM and Autodock Vina (*black*, *red* and *green curves*, respectively). *Dashed gray lines* indicate the prevalence of activity and random picking of compounds. *Vertical dashed lines* represent the thresholds we manually selected from the analysis of the curves. Metrics associated to the selected thresholds are available in Tables 2, 3, 4. Partial metrics at 2 % and 5 % of the ranked dataset are available in Additional file 1: Table S1; Additional file 2: Table S2 and Additional file 3: Table S3

**Fig. 4:**
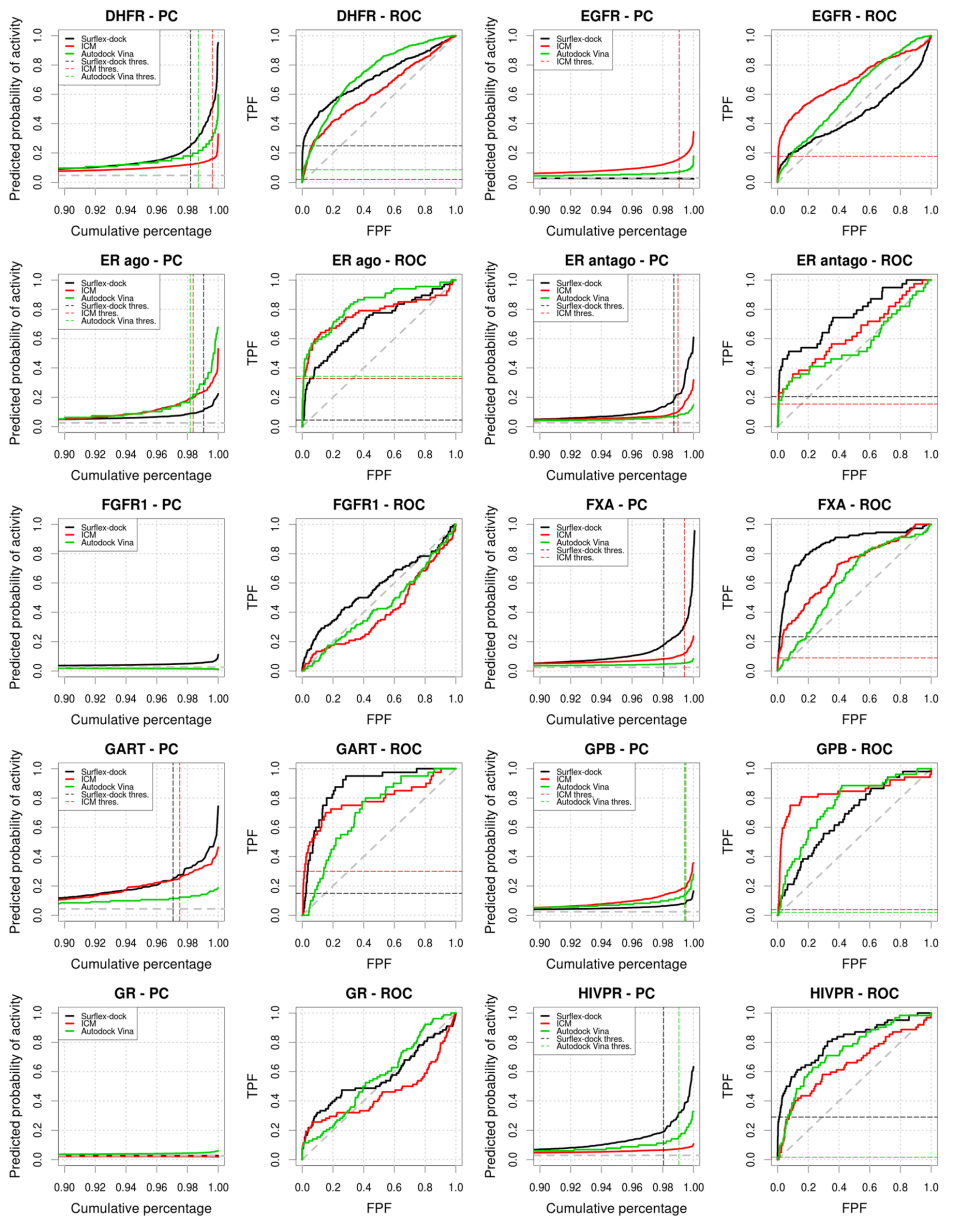
Predictiveness and ROC curves for the virtual screenings of HIVRT, HMGR, HSP90, INHA, MR, NA, P38, PARP, PDE5 and PNP selected from the DUD datasets using Surflex-dock, ICM and Autodock Vina (*black*, *red* and *green curves*, respectively). *Dashed gray lines* indicate the prevalence of activity and random picking of compounds. *Vertical dashed lines* represent the thresholds we manually selected from the analysis of the curves. Metrics associated to the selected thresholds are available in Tables 2, 3, 4. Partial metrics at 2 % and 5 % of the ranked dataset are available in Additional file 1: Table S1; Additional file 2: Table S2 and Additional file 3: Table S3

**Fig. 5:**
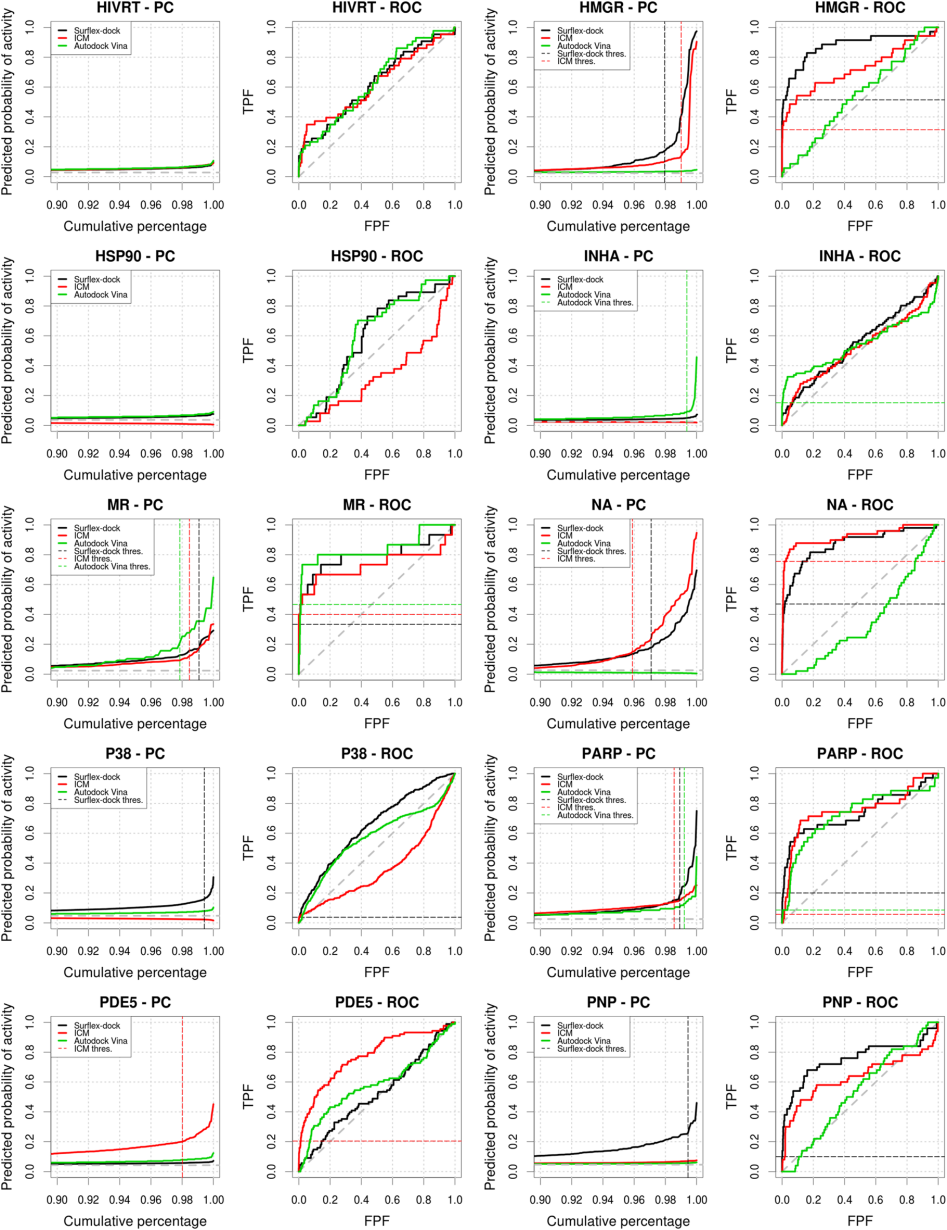
Predictiveness and ROC curves for the virtual screenings of PPAR, PR, RXR, SAHH, SRC, THR, TK, TRP and VEGFR2 selected from the DUD datasets using Surflex-dock, ICM and Autodock Vina (*black*, *red* and *green curves*, respectively). *Dashed gray lines* indicate the prevalence of activity and random picking of compounds. *Vertical dashed lines* represent the thresholds we manually selected from the analysis of the curves. Metrics associated to the selected thresholds are available in Tables 2, 3, 4. Partial metrics at 2 % and 5 % of the ranked dataset are available in Additional file 1: Table S1; Additional file 2: Table S2 and Additional file 3: Table S3

**Fig. 6:**
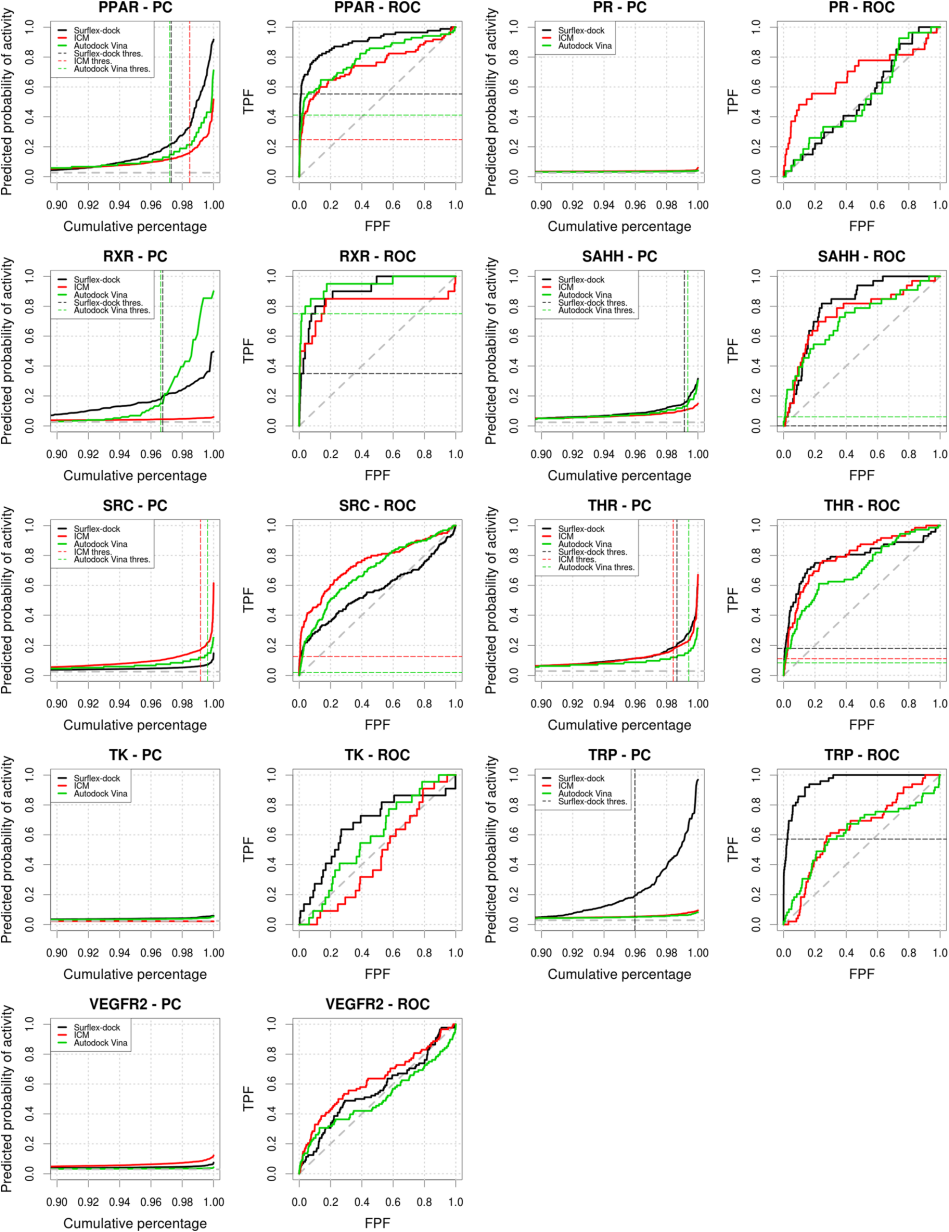
Predictiveness and ROC curves for the virtual screenings of the 39 targets we selected from the DUD datasets using Surflex-dock, ICM and Autodock Vina (*black*, *red* and *green curves*, respectively). *Dashed gray lines* indicate the prevalence of activity and random picking of compounds. *Vertical dashed lines* represent the thresholds we manually selected from the analysis of the curves. Metrics associated to the selected thresholds are available in Tables 2, 3, 4. Partial metrics at 2 and 5 % of the ranked dataset are available in Additional file 1: Table S1; Additional file 2: Table S2 and Additional file 3: Table S3

Surflex-dock, ICM and Autodock Vina screenings of the remaining datasets resulted in PCs with a profile that allowed an estimation of an optimal score selection threshold at the steepest inflexion point of the PC for respectively 22, 19 and 17 datasets. ROC AUC and TG are presented in Table 1. PCs and ROC plots are presented in Figs. 3, 4, 5 and 6 and include the display of the score selection thresholds (dashed colored lines). Score selection thresholds, pTGs, pAUCs and EFs for each virtual screening method in the resulting subsets are presented in Tables 2, 3 and 4.

**Table 1:** Description of the benchmarking dataset from the DUD, including global metrics of the virtual screens performed using Surflex-dock, ICM and Autodock Vina

Target	Nb of actives	Nb of compounds	Prevalence	Surflex-dock		ICM		Autodock vina	
				TG	ROC AUC	TG	ROC AUC	TG	ROC AUC
ACE	49	1846	0.0265	0.035	0.464	0.299	0.655	0.189	0.408
ACHE	107	3999	0.0268	0.012	0.512	0.115	0.614	0.107	0.662
ADA	39	966	0.0404	0.310	0.699	0.250	0.320	0.110	0.438
ALR2	26	1021	0.0255	0.250	0.536	0.228	0.647	0.295	0.677
AMPC	21	807	0.0260	0.227	0.687	0.134	0.534	0.214	0.325
AR	79	2933	0.0269	0.067	0.684	0.151	0.691	0.396	0.745
CDK2	72	2146	0.0336	0.186	0.608	0.364	0.734	0.212	0.620
COMT	11	479	0.0230	0.392	0.733	0.256	0.698	0.001	0.440
COX-1	25	936	0.0267	0.078	0.587	0.372	0.727	0.348	0.726
COX-2	426	13715	0.0311	0.396	0.784	0.056	0.555	0.461	0.736
DHFR	410	8777	0.0467	0.387	0.715	0.198	0.618	0.337	0.737
EGFR	475	16471	0.0288	0.018	0.461	0.352	0.697	0.159	0.605
ER ago	67	2637	0.0254	0.301	0.708	0.462	0.772	0.533	0.833
ER antago	39	1487	0.0262	0.412	0.758	0.263	0.631	0.176	0.562
FGFR1	120	4670	0.0257	0.134	0.569	0.097	0.403	0.083	0.441
FXA	146	5891	0.0248	0.521	0.860	0.326	0.702	0.132	0.616
GART	40	919	0.0435	0.555	0.881	0.492	0.783	0.287	0.710
GPB	52	2192	0.0237	0.218	0.675	0.434	0.835	0.361	0.757
GR	78	3025	0.0258	0.010	0.564	0.050	0.450	0.126	0.560
HIVPR	62	2100	0.0295	0.517	0.808	0.175	0.649	0.317	0.743
HIVRT	43	1562	0.0275	0.185	0.621	0.191	0.622	0.234	0.633
HMGR	35	1515	0.0231	0.642	0.878	0.438	0.723	0.080	0.545
HSP90	37	1016	0.0364	0.098	0.598	0.224	0.340	0.136	0.612
INHA	86	3352	0.0257	0.112	0.551	0.032	0.524	0.203	0.544
MR	15	651	0.0230	0.492	0.796	0.401	0.732	0.614	0.844
NA	49	1923	0.0255	0.633	0.870	0.764	0.923	0.198	0.350
P38	454	9595	0.0473	0.231	0.651	0.127	0.367	0.087	0.572
PARP	35	1386	0.0253	0.435	0.738	0.440	0.755	0.324	0.728
PDE5	88	2066	0.0426	0.062	0.524	0.465	0.775	0.121	0.582
PNP	50	1086	0.0460	0.404	0.755	0.072	0.635	0.034	0.536
PPAR	85	3212	0.0265	0.676	0.901	0.415	0.748	0.499	0.801
PR	27	1068	0.0253	0.109	0.527	0.130	0.686	0.080	0.525
RXR	20	770	0.0260	0.675	0.907	0.124	0.812	0.740	0.944
SAHH	33	1379	0.0239	0.391	0.811	0.330	0.751	0.338	0.717
SRC	159	6478	0.0245	0.162	0.569	0.420	0.748	0.288	0.694
THR	72	2528	0.0285	0.447	0.787	0.420	0.798	0.331	0.706
TK	22	913	0.0241	0.139	0.668	0.015	0.453	0.110	0.583
TRP	49	1713	0.0286	0.767	0.953	0.155	0.637	0.140	0.619
VEGFR2	88	2994	0.0294	0.092	0.558	0.201	0.625	0.034	0.504
Minimum	11	479	0.0230	0.010	0.461	0.015	0.320	0.001	0.325
Maximum	475	16471	0.0473	0.767	0.953	0.764	0.923	0.740	0.944
Mean	97	3134	0.0294	0.302	0.691	0.268	0.650	0.242	0.625
Median	50	1923	0.0265	0.250	0.687	0.250	0.686	0.203	0.619

The score selection thresholds for each method varied with the datasets (Surflex-dock: 6.73–12.83, ICM: −52.17 to −22.69, Autodock Vina: −12.10 to −9.00). Mean EF and median EF in the resulting subsets for each virtual screening method were superior to 13.00. The analysis thus allowed to identify target specific optimal score selection thresholds that yielded satisfying EFs, up to two digits, for 57 out of the 117 possible method/dataset associations (Figs. 3, 4, 5, 6). For 1 out of the 117 possible method/dataset associations, the defined threshold resulted in no enrichment (Surflex-dock on SAHH). For the remaining 59 method/dataset associations, the predictiveness curves suggested a defect of association between the scores obtained by the compounds and their activity.

**Table 2:** Summary of the partial metrics associated to the thresholds we selected manually from the virtual screens performed using Surflex-dock

Target	Surflex-dock—manual thresholds							
	Rank threshold	Activity threshold	pTG	pAUC	EF	Score	Actives	Cpds
ACE	–	–	–	–	–	–	–	–
ACHE	–	–	–	–	–	–	–	–
ADA	1.24	0.211	0.293	0.128	13.34	9.78	7	13
ALR2	1.18	0.103	0.119	0.231	24.17	6.73	8	13
AMPC	–	–	–	–	–	–	–	–
AR	–	–	–	–	–	–	–	–
CDK2	0.70	0.103	0.115	0.052	8.77	8.82	5	17
COMT	0.63	0.136	0.206	0.182	21.77	7.29	2	4
COX-1	–	–	–	–	–	–	–	–
COX-2	0.98	0.160	0.165	0.017	3.79	8.24	16	136
DHFR	1.80	0.251	0.389	0.207	13.73	9.36	102	159
EGFR	–	–	–	–	–	–	–	–
ER ago	0.95	0.114	0.131	0.032	4.54	8.07	3	26
ER antago	1.28	0.185	0.315	0.071	15.25	10.07	8	20
FGFR1	–	–	–	–	–	–	–	–
FXA	1.94	0.181	0.314	0.126	11.53	10.04	34	119
GART	2.94	0.249	0.345	0.062	4.92	12.47	6	28
GPB	–	–	–	–	–	–	–	–
GR	–	–	–	–	–	–	–	–
HIVPR	1.95	0.190	0.313	0.227	14.52	9.80	18	42
HIVRT	–	–	–	–	–	–	–	–
HMGR	2.05	0.173	0.471	0.449	24.35	8.91	18	32
HSP90	–	–	–	–	–	–	–	–
INHA	–	–	–	–	–	–	–	–
MR	0.92	0.172	0.227	0.200	31.00	7.32	5	7
NA	2.91	0.180	0.320	0.339	15.84	11.37	23	57
P38	0.58	0.161	0.155	0.029	6.30	8.75	17	57
PARP	1.08	0.187	0.332	0.143	17.32	7.12	7	16
PDE5	–	–	–	–	–	–	–	–
PNP	0.55	0.258	0.303	0.100	15.51	7.65	5	7
PPAR	2.71	0.221	0.441	0.395	20.18	12.83	47	88
PR	–	–	–	–	–	–	–	–
RXR	3.25	0.200	0.265	0.263	10.37	10.84	7	26
SAHH	0.87	0.154	0.200	0.000	0.00	10.08	0	13
SRC	–	–	–	–	–	–	–	–
THR	1.34	0.209	0.283	0.097	13.04	9.54	13	35
TK	–	–	–	–	–	–	–	–
TRP	4.03	0.193	0.414	0.442	13.98	8.80	28	70
VEGFR2	–	–	–	–	–	–	–	–
Minimum	0.55	0.103	0.115	0.000	0.00	6.73	0	4
Maximum	4.03	0.258	0.471	0.449	31.00	12.83	102	159
Mean	1.63	0.181	0.278	0.172	13.83	9.27	17	45
Median	1.26	0.183	0.298	0.136	13.86	9.13	8	27

**Table 3:** Summary of the partial metrics associated to the thresholds we selected manually from the virtual screens performed using ICM

Target	ICM—manual thresholds							
	Rank threshold	Activity threshold	pTG	pAUC	EF	Score	Actives	Cpds
ACE	0.49	0.14	0.166	0.136	26.37	−31.64	7	10
ACHE	–	–	–	–	–	–	–	–
ADA	–	–	–	–	–	–	–	–
ALR2	0.49	0.11	0.118	0.038	6.54	−31.46	1	6
AMPC	–	–	–	–	–	–	–	–
AR	–	–	–	–	–	–	–	–
CDK2	1.30	0.18	0.231	0.059	10.28	−29.72	10	29
COMT	–	–	–	–	–	–	–	–
COX-1	0.53	0.14	0.165	0.050	12.48	–29.72	2	6
COX-2	–	–	–	–	–	–	–	–
DHFR	0.38	0.16	0.148	0.018	5.04	−30.36	8	34
EGFR	0.93	0.16	0.179	0.101	18.79	−33.60	84	155
ER ago	1.63	0.20	0.252	0.197	19.68	−31.57	22	44
ER antago	1.01	0.10	0.157	0.062	14.30	−34.06	6	16
FGFR1	–	–	–	–	–	–	–	–
FXA	0.59	0.12	0.140	0.067	14.57	−33.80	13	36
GART	2.50	0.25	0.293	0.142	11.49	−52.17	12	24
GPB	0.50	0.19	0.240	0.010	7.03	−35.50	2	12
GR	–	–	–	–	–	–	–	–
HIVPR	–	–	–	–	–	–	–	–
HIVRT	–	–	–	–	–	–	–	–
HMGR	0.99	0.14	0.454	0.257	29.76	−26.21	11	16
HSP90	–	–	–	–	–	–	–	–
INHA	–	–	–	–	–	–	–	–
MR	1.54	0.12	0.185	0.400	23.67	−29.12	6	11
NA	4.11	0.15	0.385	0.580	18.15	−22.69	37	80
P38	–	–	–	–	–	–	–	–
PARP	1.44	0.14	0.162	0.015	3.77	−36.20	2	21
PDE5	1.98	0.20	0.232	0.151	10.06	−28.00	18	42
PNP	–	–	–	–	–	–	–	–
PPAR	1.53	0.16	0.237	0.146	15.87	−42.49	21	50
PR	–	–	–	–	–	–	–	–
RXR	–	–	–	–	–	–	–	–
SAHH	–	–	–	–	–	–	–	–
SRC	0.83	0.17	0.220	0.082	14.82	−35.14	20	55
THR	1.58	0.17	0.236	0.050	6.85	−26.85	8	41
TK	–	–	–	–	–	–	–	–
TRP	–	–	–	–	–	–	–	–
VEGFR2	–	–	–	–	–	–	–	–
Minimum	0.38	0.10	0.118	0.010	3.77	−52.17	1	6
Maximum	4.11	0.25	0.454	0.580	29.76	−22.69	84	155
Mean	1.28	0.16	0.221	0.135	14.19	−32.65	15	36
Median	1.01	0.16	0.220	0.082	14.30	−31.57	10	29

**Table 4:** Summary of the partial metrics associated to the thresholds we selected manually from the virtual screens performed using Autodock Vina

Target	Autodock Vina—manual thresholds							
	Rank threshold	Activity threshold	pTG	pAUC	EF	Score	Actives	Cpds
ACE	–	–	–	–	–	–	–	–
ACHE	–	–	–	–	–	–	–	–
ADA	–	–	–	–	–	–	–	–
ALR2	0.98	0.117	0.114	0.019	3.57	−10.10	1	11
AMPC	–	–	–	–	–	–	–	–
AR	1.06	0.141	0.214	0.113	13.92	−9.90	12	32
CDK2	0.79	0.124	0.103	0.054	13.25	−10.30	8	18
COMT	–	–	–	–	–	–	–	–
COX-1	0.64	0.165	0.225	0.000	16.05	−9.00	3	7
COX-2	1.74	0.224	0.345	0.201	19.53	−10.20	145	239
DHFR	1.29	0.218	0.250	0.051	6.57	−9.90	35	114
EGFR	–	–	–	–	–	–	–	–
ER ago	1.82	0.191	0.328	0.175	18.47	−9.50	23	49
ER antago	–	–	–	–	–	–	–	–
FGFR1	–	–	–	–	–	–	–	–
FXA	–	–	–	–	–	–	–	–
GART	–	–	–	–	–	–	–	–
GPB	0.59	0.139	0.164	0.007	3.01	−9.20	1	14
GR	–	–	–	–	–	–	–	–
HIVPR	0.95	0.159	0.192	0.012	1.61	−10.70	1	21
HIVRT	–	–	–	–	–	–	–	–
HMGR	–	–	–	–	–	–	–	–
HSP90	–	–	–	–	–	–	–	–
INHA	0.63	0.093	0.136	0.138	23.03	−11.10	13	22
MR	2.15	0.210	0.336	0.250	20.25	−10.30	7	15
NA	–	–	–	–	–	–	–	–
P38	–	–	–	–	–	–	–	–
PARP	0.79	0.130	0.160	0.048	9.90	−10.30	3	12
PDE5	–	–	–	–	–	–	–	–
PNP	–	–	–	–	–	–	–	–
PPAR	2.80	0.148	0.258	0.267	14.53	−12.10	35	91
PR	–	–	–	–	–	–	–	–
RXR	3.38	0.150	0.488	0.500	21.39	−10.60	15	27
SAHH	0.65	0.146	0.181	0.030	8.36	−9.00	2	10
SRC	0.39	0.149	0.154	0.005	4.70	−9.60	3	26
THR	0.59	0.164	0.187	0.056	13.17	−10.40	6	16
TK	–	–	–	–	–	–	–	–
TRP	–	–	–	–	–	–	–	–
VEGFR2	–	–	–	–	–	–	–	–
Minimum	0.39	0.093	0.103	0.000	1.61	−12.10	1	7
Maximum	3.38	0.224	0.488	0.500	23.03	−9.00	145	239
Mean	1.25	0.157	0.226	0.113	12.43	−10.13	18	43
Median	0.95	0.149	0.192	0.054	13.25	−10.20	7	21

The score selection thresholds for each method varied with the datasets (Surflex-dock: 6.73–12.83, ICM: −52.17 to −22.69, Autodock Vina: −12.10 to −9.00). Mean EF and median EF in the resulting subsets for each virtual screening method were superior to 13.00. The analysis thus allowed to identify target specific optimal score selection thresholds that yielded satisfying EFs, up to two digits, for 57 out of the 117 possible method/dataset associations (Fig. 3, 4, 5, 6). For 1 out of the 117 possible method/dataset associations, the defined threshold resulted in no enrichment (Surflex-dock on SAHH). For the remaining 59 method/dataset associations, the predictiveness curves suggested a defect of association between the scores obtained by the compounds and their activity.

We finally highlighted systems that illustrated the interest of using the PCs as a complement to the ROC curves: (1) Surflex-dock and ICM applied to the HMGR dataset represented one of the best-achieved early recognition cases, both PCs displaying a steep inflexion point. In this case, the analysis of the PC validated the profile of the ROC curve and informed us that the scores obtained by both methods were highly associated to the detection of active compounds; (2) For the PARP dataset, the analysis of the PCs allowed to easily estimate an optimal score selection threshold for Surflex-dock whereas ROC AUCs and ROC curve profiles were very close for all methods; (3) For the GART dataset, the PCs emphasized a better predictive performance of Surflex-dock scores over ICM’s in the early part of the dataset, whereas the ROC curves profiles could lead to an opposite interpretation of the results.

## Discussion

The goal of virtual screening methods in drug discovery programs is to predict the potential activity of the compounds of a compound collection on a specific target. The result is a list of compounds ranked by a scoring function that estimates the activity on the target (binding affinity, equilibrium constant, binding energy), which will be confirmed experimentally. Since scoring functions are still the most limiting factor in virtual screening in particular to predict activity, it is usual to select empirically the top scoring compounds for experimental tests [27–29]. Several performance metrics were developed over the years to evaluate the performance of virtual screening methods and guide the definition of the best protocols. The most used metrics suffer from three main limitations; (1) they focus on the predicted ranks of the compounds according to the scoring function instead of taking into account the value of the score; (2) they do not focus particularly on the top scoring compounds; (3) they do not allow an intuitive estimation of the score threshold that would give the best confidence into finding active compounds. In the present work, we suggested the use of a metric that tackles these limitations, the Predictiveness Curve.

As expected, the score values issued from scoring functions differ from one system to another rendering direct score comparisons between different systems difficult. That is why benchmarking metrics use specificity and selectivity to focus on the ranks of the compounds according to the scoring functions instead of the score values. In prospective virtual screening experiments, since score values and resulting ranks are available to the expert, both should be used to perform the compounds selection for experimental tests. As pointed out by Triballeau et al., a ROC AUC of 0.9 means that a randomly selected active molecule has a higher score than a randomly selected inactive 9 times out of 10 [2]. However, it does not mean that a hit would be confirmed experimentally with a probability of 0.9. ROC curves characterize the overall inherent quality of a virtual screening experiment and by no means are indicative of the quality of a particular compound or of a given subset of the initial compound collection. Finally, ROC plots do not allow a direct estimation of the size of an optimal subset in terms of activity potential, which is a critical task of virtual screening. We suggested in the present work the use of logistic regression and PC analysis to provide activity probabilities related to the scores obtained by the compounds after virtual screening.

Considering early recognition, it seems surprising that in other fields where this problem occurs, such as information retrieval, the metrics that are commonly used are not particularly efficient [30]. Likewise, there is still no consensus on the optimal metric to use to analyze the performance of virtual screening methods. ROC and EF are not able to discriminate the “ranking goodness” before the fractional threshold [4]. Furthermore, if two ranked lists display similar initial enhancements, but differ significantly just after the selection threshold, they would not be differentiated using EF or partial ROC metrics [2, 4, 31]. Since the overall distribution of the scores after virtual screening is taken into account by predictiveness models, the PC is able to perform efficient differentiation in this case. Hence, by summarizing the PC over a restricted range of compounds, pTG quantifies the enhancement of activity in the early part of the ranked molecular dataset and is a function of the overall success of the virtual screening experiment [20].

Now considering the choice of score selection thresholds towards prospective virtual screening experiments, Neyman and Pearson, who pioneered hypothesis testing, asserted that there is no general rule for balancing errors [32]. In any given case, the determination of “how the balance [between wrong and correct classifications] should be struck, must be left to the investigator” [32]. In summary, balancing false-positive and false-negative rates has “nothing to do with statistical theory but is based instead on context-dependent pragmatic considerations where informed personal judgment plays a vital role” [33]. Triballeau et al. transferred the ROC curve to the field of virtual screening and described how to retrieve score thresholds by maximizing either specificity or sensitivity from the ROC analysis [2]. The PC has the advantage to provide a probability-related interpretation of the scores by taking into account their variations, which efficiently complements the ROC curve for benchmarking purposes. Predictiveness curves allow for the detection of optimal score selection thresholds in an intuitive and straightforward way; a task for which the ROC curves are not adapted. Through the analysis of PCs, we were able to estimate optimal score selection thresholds for each virtual screening method used in the study, which were associated to satisfying EFs in each resulting subset. We were also able to detect an absence of association between the scores obtained by the compounds after virtual screening and the activity of the compounds, in particular for experiments that yielded high ROC AUC values. We demonstrated these usages on the DUD dataset for three virtual screening methods, providing all PC and ROC curves with scores and metrics associated to each resulting subset (Figs. 3, 4, 5, 6; Tables 2, 3, 4).

The first objective of this paper is to introduce to the field of virtual screening the predictiveness curves for the purpose of benchmarking retrospective virtual screening experiments. We believe that benchmarking metrics have to take into account the values of the scores calculated in a virtual screening experiment for a better understanding of its results; which may also support the enhancement of the performances of scoring functions. The second objective of this paper is to provide a method to define score selection thresholds to be used for prospective virtual screenings, in order to select an optimal number of compounds to be tested experimentally in drug discovery programs. The predictiveness curves graphically emphasize the differences in scores that are relevant for the detection of active compounds in a virtual screening experiment and ease the process of defining optimal thresholds. When retrospective studies on a specific target allowed to detect optimal score selection thresholds, considering that a prospective virtual screening experiment could be performed under similar conditions, we can expect score variations to be reproducible and the corresponding score thresholds to be transferable. Therefore, the resulting subset of compounds selected when applying the estimated score threshold would be expected to be highly enriched in active compounds. However, score selection thresholds defined in retrospective studies must be considered carefully when applied for the selection of molecular subsets in prospective studies. It is important to keep in mind that all performance measures should be interpreted in the context of the composition of the benchmarking datasets [34, 35] and that the score selection thresholds that would be estimated during the benchmark should be adapted to the composition of the dataset that will be used for prospective screening.

## Conclusion

The value of a continuous test in predicting a binary outcome can be assessed by considering two aspects: discrimination and outcome prediction. In the present study, we proposed predictiveness curves as a complement to the existing methods to analyze the results of virtual screening methods. Logistic regression models can be used to evaluate the probability of each compound to be active given the score it obtained through the virtual screening method. The PC then provides an intuitive way to visualize the data and allows for an efficient comparison of the performance of virtual screening methods, especially considering the early recognition problem. Performance metrics are easily estimated from the predictiveness plots: TG, pTG, PPV, NPV, TPF and NPF. PC also ease the process of extracting optimal score selection thresholds from virtual screening results, which is a valuable step to proceed to prospective virtual screening. The enhancement of activity attributed to the variations of virtual screening scores can then be quantified in the resulting subsets of compounds using the pTG.

Visualizing both the predictiveness curve and the ROC curve empowers the analysis of virtual screening results. The two measures, however, summarize different aspects of the predictive performance of scores and thus answer different questions [14, 20]. On the one hand, we are interested in the ROC curve because it summarizes the inherent capacity of a virtual screening method to distinguish between active and inactive compounds. This information would aid in the decision to whether or not apply a virtual screening method in the first place. On the other hand, the predictiveness curve informs us on the association between virtual screening scores and the activity of the compounds. This information would aid in decision making when performing prospective virtual screening experiments. By simultaneously displaying PC and ROC, we believe researchers will be better equipped to analyze and understand the results of virtual screening experiments.

## Additional files

10.1186/s13321-015-0100-8 Summary of the partial metrics at 2 % and 5 % of the ordered dataset for virtual screens performed using Surflex-dock.
10.1186/s13321-015-0100-8 Summary of the partial metrics at 2 % and 5 % of the ordered dataset for virtual screens performed using ICM.
10.1186/s13321-015-0100-8 Summary of the partial metrics at 2 % and 5 % of the ordered dataset for virtual screens performed using Autodock Vina.

## Abbreviations

VS: virtual screening
PC: predictiveness curve
EF: enrichment factor
ROC: receiver operating characteristic
AUC: area under the curve
pAUC: partial AUC
BEDROC: Boltzmann-enhanced discrimination of ROC
RIE: robust initial enhancement
AUAC: area under the accumulation curve
TPF: true positive fraction
FPF: false positive fraction
TG: total gain
pTG: partial total gain
DUD: directory of useful decoys
CDF: cumulative distribution function
ACE: angiotensin-converting enzyme
ACHE: acetylcholin esterase
ADA: adenosine deaminase
ALR2: aldose reductase
AMPC: AmpC beta lactamase
AR: androgen receptor
CDK2: cyclin dependent kinase 2
COMT: catechol O-methyltransferase
COX-1: cyclooxygenase-1
COX-2: cyclooxygenase-2
DHFR: dihydrofolate reductase
EGFR: epidermal growth factor receptor kinase
ER: agoestrogen receptor agonist
ER: antagoestrogen receptor antagonist
FGFR1: fibroblast growth factor receptor kinase
FXA: factor Xa
GART: glycinamide ribonucleotide transformylase
GPB: glycogen phosphorylase beta
GR: glucocorticoid receptor
HIVPR: HIV protease
HIVRTHIV: reverse transcriptase
HMGR: hydroxymethylglutaryl-CoA reductase
HSP90: human heat shock protein 90 kinase
INHA: enoyl ACP reductase
MR: mineralocorticoid receptor
NA: neuraminidase
P38: P38 mitogen activated protein kinase
PARP: poly(ADP-ribose) polymerase
PDE5: phosphodiesterase V
PDGFR-β: platelet derived growth factor receptor kinase beta
PNP: purine nucleoside phosphorylase
PPAR: peroxisome proliferator activated receptor gamma
PR: progesterone receptor
RXR: retinoic X receptor alpha
SAHH: S-adenosyl-homocystein hydrolase
SRC: tyrosine kinase SRC
THR: thrombin
TK: thymidine kinase
TRP: trypsin
VEGFR2: vascular endothelial growth factor receptor kinase
NR: nuclear receptors
