# Supplementary material for: Predictiveness curves in virtual screening
Source: J Cheminform. 2015 Nov 4;7:52. doi: 10.1186/s13321-015-0100-8 (PMC4631717; doi:10.1186/s13321-015-0100-8)
Supplement: Supplementary file 3 — 10.1186/s13321-015-0100-8 Summary of the partial metrics at 2 % and 5 % of the ordered dataset for virtual screens performed using Autodock Vina. [file 13321_2015_100_MOESM3_ESM.docx]

**Additional table 3.** Summary of the partial metrics at 2% and 5% of the ordered dataset for virtual screens performed using Autodock Vina.
